# Prevalence of the molecular marker of *Plasmodium falciparum* resistance to chloroquine and sulphadoxine/pyrimethamine in Benin seven years after the change of malaria treatment policy

**DOI:** 10.1186/1475-2875-12-147

**Published:** 2013-05-01

**Authors:** Aurore Ogouyèmi-Hounto, Nicaise Tuikue Ndam, Dorothée Kinde Gazard, Sitou d’Almeida, Lucette Koussihoude, Elvire Ollo, Carmine Azagnandji, Mourchidath Bello, Jean-Phillipe Chippaux, Achille Massougbodji

**Affiliations:** 1 Faculté des Sciences de la Santé, Laboratoire du centre de lutte intégrée contre le paludisme, Cotonou 01 BP188, Benin; 2 Institut de Recherche pour le Développement, Cotonou 08 BP 841, Benin

**Keywords:** Prevalence-mutation, *P. falciparum*, Chloroquine, Sulphadoxine-pyrimethamine

## Abstract

**Background:**

In Benin, the National Malaria Control Programme (NMCP) changed the policy of malaria treatment in 2004 following increasing of failure rate of treatment with chloroquine (CQ) and sulphadoxine-pyrimethamine (SP). The objective of this study was to determinate the prevalence of *Plasmodium falciparum* molecular markers that are associated with resistance to CQ and SP in Benin seven years after the new policy was instituted.

**Methods:**

The study was conducted in southern Benin, a region characterized by a perennial malaria transmission. Blood samples were collected in 2011 from children presenting with symptomatic and asymptomatic *P. falciparum* infections and living in the same area. The prevalence of critical point mutations in the genes of *pfcrt* (codon 76), *pfmdr1* (codon 86), *pfdhfr* (codons, 51, 59 and 108) and *pfdhps* (codons 437, 540) was examined in parasite isolates by mutation-specific restriction enzyme digestion of nested PCR products.

**Results:**

A high prevalence of parasites carrying point mutations in all studied targets was found: T*76:* 93.9% [89.8; 96.7], I*5*1: 96.2% [92.7; 98.4], R59*:* 93, 9% [89.7; 96.7], N108*:* 97.6% [94.6; 99.2] and G437: 71.4% [64.8; 77.4]. No mutation was found at codon 540 of the *pfdhps* gene. The proportion of parasite isolates carrying triple mutation in the *pfdhfr* gene *IRN* (I*5*1, R59 andN108) and quadruple mutation on the combination of *pfdhfr/pfdhps* IRNG (I*5*1, R59, N108 and G437) was 91.5% [86.9; 94.9] and 65.7% [58.9; 72.1], respectively. Analysis of mutation in relation to the clinical status (symptomatic or asymptomatic) and according to age (younger or older than 10 years) showed similar very high frequencies in each category without significant difference between two groups.

**Conclusions:**

These results suggest a persistence level of resistance of *P. falciparum* to CQ and SP, seven years after the recommendation of the change of malaria treatment policy in Benin. The distribution of mutations studied was neither related to age nor to clinical status.

## Background

In Benin, the anti-malarial treatment policy has long been based on the use of chloroquine (CQ) and sulphadoxine-pyrimethamine (SP) as first- and second-line treatments, respectively. *In vivo* efficacy studies, conducted in 2002 by the National Malaria Control Programme (NMCP) according to the WHO protocol, had revealed treatment failures rates by region ranging from 15.0–61.3% with CQ and 3.3–45.9% with SP in under-fives followed up to 14 days [1]. These studies were performed in five regions of the country located in the south (Lokossa), centre (Dassa Zoume, Abomey) and north (Kouandé, Malanville), with an overall failure rate of 35.2 for CQ (11.5% and 23.7% early and late treatment failures respectively) and 22.8% for SP (8.3% and 24.5% early and late treatment failures respectively) (unpubl. data from NMCP, Ministry of Health). Based on these observations, the national anti-malarial drug policy was changed in 2004 by the official withdrawal of CQ and SP in the treatment of uncomplicated malaria. However, SP is still used for the prevention of malaria in pregnancy through intermittent preventive treatment (IPTp), as recommended by WHO. The current policy is based on the use of artemether-lumefantrine (AL)(Coartem®) and artesunate amodiaquine (ASAQ)(Arsucam®) as first-line treatment for uncomplicated malaria. It should be noted that between 2004 and 2005 there was an overlap of treatments with CQ, SP and AL, ASAQ, due to the unavailability of the new treatment in some health facilities. In 2005, Aubouy [2] conducted an *in vivo* study according to the WHO protocol over 28 days [3], which showed very high failure rates (85.7% and 50%), to CQ and SP, respectively. Since the first reports pointing the decreased efficacy of anti-malarial drugs on malaria parasites in endemic regions, important investigations led to the discovery of the involvement of genetic mutations in these parasites conferring resistance [4,5]. Thus, mutations occurring on the gene *pfcrt* (T76), and secondarily *pfmdr1* (Y86), *pfdhps* (G437, E 540, A436), *pfdhfr* (I51, R59, N108,) have respectively been associated with resistance of *P. falciparum* to CQ, and SP [6-9]. Moreover, it has been increasingly reported in some African settings, that *P*. *falciparum* parasites again become susceptible to CQ several years after the withdrawal of the molecule [10,11]. In Benin, no data on the prevalence of parasite molecular markers of resistance was available before the treatment policy change and only a limited number of studies of molecular markers of anti-malarial drug resistance in *P. falciparum* have been carried out [12,13]. These studies have focused on genes *pfdhfr* and *pfdhps* and reported high proportion of quadruple mutant parasites (above 80%). Thus, it seemed appropriate to assess the prevalence of different markers of resistance to CQ and SP in the population of Benin seven years after the official withdrawal of those drugs in the treatment of malaria in Benin. The main objective of this study was to determine the prevalence of *P. falciparum* molecular markers that are associated with resistance to CQ and SP by analysing the point mutations in *pfcrt, pfmdr1, pfdhfr* and *pfdhps* gene using samples from asymptomatic children and those with uncomplicated malaria in southern Benin.

## Methods

### Study sites and population

The study was conducted in two highly endemic regions of southern Benin, including the departments of Littoral and Ouémé. Southern Benin is characterized by a sub-equatorial climate and a perennial malaria transmission with two peaks corresponding to the rainy seasons (April–July and mid-September–November) [14]. Children aged between six months and 15 years, who resided in the study sites for more than a period of six months were enrolled from May through August 2011. Asymptomatic children were recruited among the nursery and primary school pupils in the study area when they showed a positive thick smear regardless of parasite density. A census of all nurseries and primary schools in the study area was initially conducted, from which a draw of five nurseries and five primary schools was made. In each school included, all attending students in all open classes on the day of the survey were involved in the study. The age ranged from 3 to 5 years in nurseries and 6 to 18 years in primary schools (children over 15 years were screened to evaluate the carriage rate of *P. falciparum* in the study area, but were removed from the sample for molecular analysis). Written informed consent from the principal and parents were obtained. For the selection of symptomatic children, WHO protocol for *in vivo* studies was applied to avoid severe malaria or uncomplicated malaria associated with other diseases. Thus, children visiting the health facilities in the study area and who met the criteria below were enrolled in the study: (i) fever (axillary temperature ≥ 37.5°C) or a history of fever within the past 48 hours, (ii) *P. falciparum* mono-infection with parasite density ≥ 1,000 asexual forms per microlitre, identified by microscopy on blood smears; (iii) no evidence of a concomitant febrile illness; iv) no sign/symptoms of severe malaria as defined by WHO [15] and (v) written informed consent from parents.

### Sample collection and laboratory procedure

Venous blood from symptomatic children fulfilling the above criteria was collected systematically on the filter paper. Samples of asymptomatic children containing parasites were also stored as spots on filter paper. Thick and thin blood smears were prepared and were stained with 10% Giemsa for rapid diagnostic. All thick blood smears were examined against 500 leucocytes. Parasite densities were recorded as the number of parasites/μl of blood, assuming an average leukocyte count of 8,000/μl of blood. All slides were read in the laboratories of the health centres, with external quality control performed on 10% of the negative slides and all positives in the reference Parasitology Laboratory of the Centre National Hospitalo-Universitaire in Cotonou. All malaria-infected patients, based on microscopy results, were treated according to malaria treatment policy based on ACT: artemether/lumefantrine.

### DNA extraction, PCR- RFLP assay

Parasite DNA was extracted using the Chelex methods [16] and stored at −20°C until use. The regions of the *pfcrt, pfmdr1*, *pfdhfr* and *pfdhps* genes containing the polymorphisms of interest were amplified by nested PCR described previously [17-19]. The RFLP was performed by digesting 6 μl (*pfcrt* and *pfmdr1*) or 10 μl (*pfdhfr* and *pfdhps*) of PCR products with specific restriction enzymes (that varied according to the studied codons) and buffers obtained from New England Biolabs (Medinova, Glostrup, Denmark) according to supplier’s specifications. The *pfcrt* mutation at codon 76, *pfmdr1* mutation at codon 86, *pfdhfr* mutations at codons 51, 59, 108, and *pfdhps* mutations at codons 437 and 540, were characterized. Five laboratory clones (3D7, FCR3, HB3, D10 and Dd2) used as a standard positive controls and negative controls (without DNA) were amplified and also digested with the samples. The digested products were visualized by electrophoresis on 1.5% agarose gels containing ethidium bromide. Gels were recorded by digital photography. Four samples of each series were randomly selected and tested repeatedly to check the reproducibility of the technique. Enzymes and control used, the size of the fragments obtained after cleavage of mutant or wild-type strains are shown in Table 1. Control strains were used for each analysis and mutations were determined to be present or absent on the basis of the presence or absence of the expected bands on the gel. Markers of 100 bp (Pharmacia Biotech) were used to size the bands. Each codon was characterized as wild-type (no mutation present), pure mutant (only mutant genotypes detected). Cases of mixed infection (wild type and mutant) were categorized as mutant throughout the analysis. All molecular analyses were performed in the Molecular Biology Laboratory of Center for integrated malaria control (CLIP).

**Table 1:** Enzymes and control used, number and size of fragments obtained by codon

**Restriction enzymes**	**Wild type alleles/control used**	**Number and size of bands obtained (bp)**	**Mutant alleles/control used**	**Number and size of bands obtained (bp)**
***pfdhfr : *Nested PCR product size : 594**				
Alu I	S108/3D7	2bands : 280+ 314	N108 or T108 HB3, Dd2	1 band : 594
Tsp 509 I	N51/3D7 FRC3	2 bands : 125+ 150	I51/Dd2	2bands : 125+ 250.
Xmn I	C59/3D7, FCR3	2bands : 250+ 344	R59/Dd2	2bands : 250+ 320
***pfdhps : *Nested PCR product size : 711**				
Ava II	A437/FCR3	1band : 711	G437/3D7, Dd2	1band : 650
Fok I	K540/3D7, Dd2, FCR3	1band : 711	E540/D10	2bands : 311 + 400pb
***pfcrt : *Nested PCR product size : 145**				
Apo I	K76/3D7, HB3	2 bands : 99 + 46	T76/Dd2	1 band : 145
***pfmdr1 : *Nested PCR product size : 521**				
AFI III	N86/3D7, HB3	1 band : 521	Y86/FCR3	2 bands : 346 + 175

### Data analysis

The data were entered in the software Reversion 2.12.0 (R Foundation for Statistical Computing, Vienna, Austria). The frequency of a particular mutant was calculated as the proportion of the specific mutant samples among the total number of samples successfully analysed for this mutation. Similarly, the frequencies of double, triple and quadruple mutants were determined as the proportion of subjects with two, three and four mutations among the total numbers of samples tested for the each. To investigate the relationship between the mutation and age, children were segregated into two categories: children below and above 10 years of age. The reason for this division is that recent intensification of malaria control activities in the country, such as the widespread distribution and use of insecticide-treated nets, large-scale indoor residual spraying is likely to impact the acquisition of immunity, usually achieved at about five years of age in endemic areas [20]. Wilcoxon test and Student test were used to compare the distribution of age according to the clinical status and distribution of the parasite density respectively. The chi-square test or Fisher’s exact test was used for proportion comparisons. The significance level (P < 0.05) was used to make the link between mutations, clinical status and age.

### Ethical approval

This study was approved by Ethical Committee of the Faculté des Sciences de la Santé, Cotonou, Benin.

## Results

### Characteristics of the study population

A total of 2,249 asymptomatic children were screened for malaria parasitaemia; 208 samples were found positive for *P. falciparum* and were collected and stored as spots on filter paper. The carriage rate of *P. falciparum* was 9.2%. From these positive cases, 143 samples were selected for molecular analysis (after the withdrawal of children above 15 years age). A total of 73 samples of symptomatic subjects were collected and analysed. Thus a total of 216 samples were analysed for point mutations. The characteristics of subjects enrolled are shown in Table 2. For each target studied, the number of isolates actually analysed depended on the success of PCR amplification and were 213 for *pfcrt*, 212 for *pfmdr1*and *pfdhfr*, and 210 for *pfdhps*. Retested samples to check the reproducibility results were consistent with those found initially.

**Table 2:** Characteristics of the study population

		**Symptomatic children**	**Asymptomatic children**	**p-value**
Number (%)		73 (33.8%)	143 (66.2%)	
Age (years)	Mean (SD)	7.8 (3.0)	8.3 (2.5)	
	Median (IQR)	8 (7–9)	9 (8–10)	0.09^w^
Children below 10 years n (%)		56 (76.7%)	82 (57.3%)	0.008^b^
M/F ratio		39/34; 1.15	73/70; 1.04	0.85^b^
Parasitaemia	Geometric mean 95% CI	6822.58	656.69	<0.001^t^
		[5072.29; 9176.86]	[474.74; 908.36]	
	Median (IQR)	7000 (2400–15000)	1000 (150–2520)	<0.001^w^

w: Wilcoxon’s test; b: Chi 2 test t: Student test.

### Prevalence of *pfcrt* and *pfmdr1,* alleles and mutations

**Figure 1:**
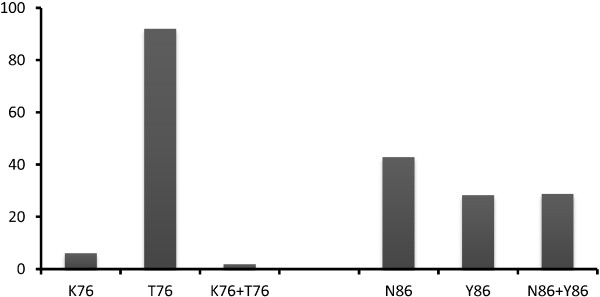
**Prevalence of *pfcrt *and *pfmdr *alleles.**

The mutant allele T76 of *pfcrt* gene was present in 92% (196/213) of samples from patients that were analysed for this mutation. In contrast, only 28.3% (60/212) of samples analysed carried mutation type allele Y86 of *pfmdr1.* 42.9% carried the wild-type allele (N86), and 28.8% carried a mixture of the wild type and the mutant allele (N86Y86) (Figure 1).

The T76 mutation was present in 93.9% (200/213) [89.8; 96.7], which was higher in that population than Y86: 57.1% (121/212) [50.1; 63.8] and the double mutation T76Y86: 55.2% (117/212) [48.5; 62.3] (P < 0.001) (Table 3).

**Table 3:** **Prevalence of molecular markers associated with *P. falciparum *resistance to CQ and SP in symptomatic and asymptomatic children**

**Molecular marker**	**Population n (%)**	**Symptomatic n (%)**	**Asymptomatic n (%)**	**p-value**
T76 (n = 213)	200 (93.9%)*	68/72 94.4%)	132/141 (93.6%)	1^a^
Y86 (n = 212)	121 (57.1%)	29/71 (40.8%)	92/141 (65.2%)	0.03^b^
T76Y86 (n = 212)	117 (55.2%)	28/71 (39.4%)	89/140 (63.6%)	0.03^b^
G437 (n = 210)	150 (71.4%)	48/73 (65.8%)	102/137 (74.5%)	0.42^b^
E540 (n = 210)	0%	0%	0%	
I51 (n = 212)	204 (96.2%)	69/73 (94.5%)	135/139 (97.1%)	0.45^a^
R59 (n = 212)	199 (93.9%)	69/73 (94.5%)	130/139 (93.5%)	1^a^
N108 (n = 212)	207 (97.6%)	71/73 (97.3%)	136/139 (97.8%)	0.72^a^
IRN^1^ (n = 212)	194 (91.5%)	67/73 (91.8%)	127/139 (91.4%)	0.61^b^
IRNG^2^ (n = 210)	138 (65.7%)	44/73 (60.3%)	94/137 (68.6%)	0.15^b^

^1^*pfdhfr* triple mutants (I51, R59, and N108).

^2^*pfdhfr/pfdhps* quadruple mutants (*pfdhfr* I51, R59, N108 and *pfdhps* G437).

*p < 0.001 of Chi 2 test (comparison between T76 and Y86, T76 and T76Y86).

^a^ p-value of fisher test.

^b^ p-value of Chi 2 test.

### Prevalence of *pfdhps* and *pfdhfr* alleles and mutations

**Figure 2:**
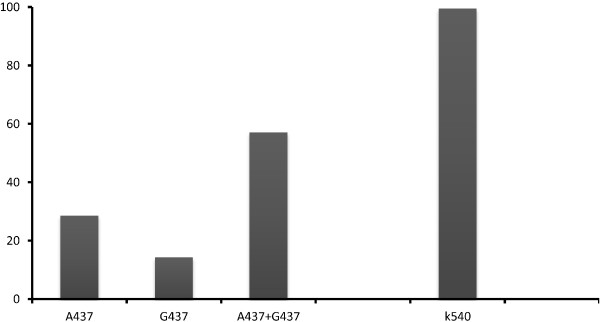
**Prevalence of *pfdhps *alleles.**

***Pfdhps:*** a mixture of the wild type and the mutant allele (A437G437) was more represented with codon 437: 57.1% (120/210), whereas all samples carried wild type allele (K540) with codon 540 (Figure 2). Mutation of codon 437 (*G*437*)* was high with 71.4% (150/210) [64.8; 77.4] while no mutation was found with the codon 540 (E540) in the study population (Table 3).

**Figure 3:**
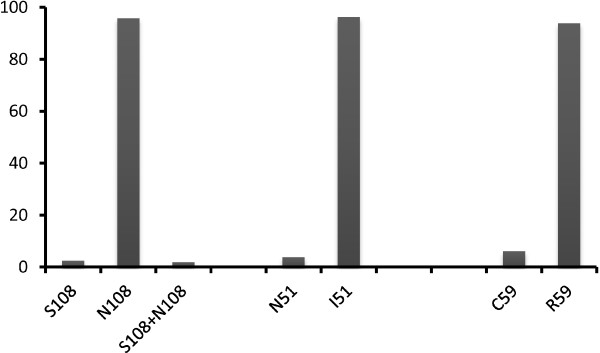
**Prevalence of *pfdhfr *alleles.**

***Pfdhfr:*** mutated alleles were more represented for codon 51 (I51), codon 59 (R59) and codon 108 (N108), respectively, 96.2% (204/212), 93.9% (199/212), and 95.8% (203/212). No mixture of alleles was found with codon 51 and codon 59, but in a very small proportion with codon 108 (S108N108): 1.9% (4/212) (Figure 3).

The proportion of single, triple and quadruple mutation was very high in the study population: I51: 96.2% (204/210) [92.7; 98.4], R59: 93.9% (199/212) [89.7; 96.7], N108: 97.6% (207/212) [94.6; 99.2], *Pfdhfr* triple mutant IRN (I51, R59 and N108): 91.5% (194/212) [86.9; 94.9], *Pfdhfr/Pfdhps* quadruple mutant IRNG (*Pfdhfr* I51, R59, N108, and *Pfdhps* G437): 65.7% (138/210) [58.9; 72.1] (Table 3).

### Mutation and clinical status and age

According clinical group, Y86 and double mutation T76Y86 were more represented in asymptomatic group. P = 0.03. Otherwise, there was no significant difference between prevalence of single, double, triple or quadruple mutation and clinical status (Table 3). When the data were analysed by age categories similar frequencies of single, triple and quadruple mutant parasite were found in children younger than 10 years and in older children, regardless of the clinical status. The purpose here was to see if the distribution of mutant parasites could be affected by age and therefore probably by the gradual acquisition of immunity. However, a tendency towards elevation of Y86 and double mutation T76Y86 rate among symptomatic children less than 10 years was noted but no statistically significant difference (Table 4).

**Table 4:** **Prevalence of mutations conferring resistance to CQ and SP in *Plasmodium falciparum *according age**

**Molecular marker**	**Symptomatic**		**Asymptomatic**	
	**< 10 ans**	**≥ 10 ans**	**< 10 ans**	**≥ 10 ans**
*T* 76	53 (94.6%)	15 (93.8%)	74 (91.4%)	58 (96.7%)
Y86	25 (45.5%)	4 (25%)*	49 (60.5%)	43 (71.7%)
T76 + Y86	24 (43.6%)	4 (25%)**	47 (58.8%)	42 (70%)
G437	36 (64.3%)	12 (70.6%)	54 (68.4%)	48 (82.8%)
E540	0%	0%	0%	0%
I51	52 (92.9%)	17 (100)	77 (96.2%)	58 (98.3%)
R59	53 (94.6%)	16 (94.1%)	77 (96.2%)	53 (89.8%)
N108	54 (96.4%)	17 (100)	78 (97.5%)	58 (98.3%)
IRN^1^	51 (91.1%)	16 (94.1%)	76 (95%)	51 (86.4%)
IRNG^2^	33 (58.9%)	11 (64.7%)	52 (65.8%)	42 (72.4%)

^1^*pfdhfr* triple mutants: (I51, R59, and N108).

^2^*pfdhfr/pfdhps* quadruple mutants: (*pfdhfr* I51, R59, N108 and *pfdhps* G437).

*p = 0.16 (fisher test).

**p = 0.24 (fisher test).

## Discussion

The main objective of this study was to evaluate the prevalence in Benin of *P. falciparum* resistance markers to CQ and SP, two conventional anti-malarials that have been used for a long time. This study was justified by the need of surveillance of *P. falciparum* resistance to anti-malarials drugs in Benin through well-characterized molecular markers of resistance to contribute to the monitoring in the subregion. This monitoring is intended on the one hand to watch for a possible return of sensitivity of *P. falciparum* to CQ and secondly, to describe the current patterns of molecular markers associated with parasite resistance to SP, the latter still used as a preventive treatment of malaria in pregnant women. This work was carried out on parasites obtained from two distinct clinical groups of children, to investigate a possible relationship between molecular markers and clinical status. Mutation at codon 164 of *pfdhfr* gene has not been studied because several studies have noted the absence or rarity of this mutation in African isolates [13,21-24].

High rates of single, double, triple or quadruple mutation observed in this study reflect the current level of sensitivity of *P. falciparum* to CQ and SP in Benin, which is still expected to be low although the two drugs were officially withdrawn from curative treatment of uncomplicated malaria in 2004. These high rates of mutant parasites had been previously found by other authors [23,25-28]. The fact that the frequency of mutations at codon site 437 of *pfdhps* is lower than those observed on the *pfdhfr* gene confirms that the mutations associated with parasite resistance to SP appeared earlier on the *pfdhfr* than those affecting the *pfdhps*[29,30].

Similar to other reports from the sub-region [31,32], the parasites harbouring the mutation at codon 540E were not found in Benin. Regarding the *pfcrt* gene, analysis of the T76 mutation (93.9%) in isolates from Benin showed a high prevalence of parasites carrying this mutation. Unlike Malawi, where there has been a reversal of the prevalence of mutant T76 a few years after the withdrawal of CQ [10,11], it is clear that this has not been the case in Benin. Self-medication with fake medicines especially with regard to CQ (despite its formal withdrawal from the treatment policy of malaria) could be a leading cause for maintaining this high prevalence of T76 mutant parasites as a result of continued and frequent use of CQ [33] involving either insufficient doses or too short a duration of administration. It is a common practice in the south of the country characterized by large markets (with neighbouring Nigeria) where illegitimate distribution of fake drugs is common [2,34]. This could also be marginally explained by a possible cross-resistance shared between CQ and AQ [35,36], since AQ is present in the ASAQ combination currently used for malaria management in Benin. WHO *in vivo* drug efficacy studies conducted in 2008 with this combination in two localities of the country noted an adequate clinical and parasitological response (ACPR) of 100% and 76.6% respectively without PCR correction. The second locality (Dassa Zoume) with a decrease of ACPR (23.44% of late parasitological failure) had posted 38.8% of late parasitological failure in 2002 during in vivo drug efficacy studies with CQ (unpublished data from Ministry of Health, Benin).

Thus, the re-emergence of sensitive parasite strains after the withdrawal of CQ depends not only on the time elapsed since the withdrawal of the treatment, but also on the speed of replacement and implementation of the new drugs, to allow the cessation of use and consumption of drugs replaced. It is important that the health authorities of the country ensure the effective withdrawal of CQ by emphasizing the education about the risks of self-medication and implementing control methods against the entry of fake medicines. The results obtained with the gene *pfcrt* are consistent with those reported in China by Zang *et al.*[37], but is in contrast to those reported by Kamugisha *et al.* in Tanzania [38] and Raman *et al.* in Mozambique [39].

Regarding SP, it should be noted that the use of other sulpha drugs, such as trimethoprim-sulphametoxazole (co-trimoxazole) often prescribed for bacterial infections in children but also in prevention and treatment of opportunistic infections in persons living with HIV, contribute in maintaining drug pressure on malaria parasites. The use of SP in intermittent preventive treatment in pregnant women (IPTp) since its withdrawal from the treatment of uncomplicated malaria may also explain the maintain of the selective pressure on SP targets as shown in the study conducted by Bertin *et al.* in Benin and Pearce *et al.* in Tanzania [13,40]. Similarly to what has been reported by other authors [17,27], this study shows that regardless of the gene, mutations are not associated with the clinical status of children. On the other hand, to determine whether immunity contributed to the ability to clear infections by parasites carrying resistance, the proportion of infections by parasites carrying different mutations compared between children younger than 10 years and older children suggests that age does not influence the distribution and carriage of resistant parasites whatever the clinical status and type of mutation.

## Conclusions

This study showed high prevalence of *pfcrt76* and quadruple *phdhfr/pfdhps* mutants parasites (triple mutant *pfdhfr* + single 437 *pfdhps* mutant) confirming the persistence of parasite resistance to CQ and SP in Benin several years after officially withdrawal of these two drugs in the treatment of uncomplicated malaria. Clinical status and age do not seem to influence the frequency of individual mutants or the distribution of the triple and quadruple mutant parasites. This study has generated data which will be useful in monitoring the dynamics of drug resistant malaria parasites in Benin and in the sub-region.

## Competing interests

The authors declare that they have no competing interests.

## Authors’ contributions

DKZ designed the study protocol, supervised the study and corrected the manuscript; AOH participated in the design of study, supervised the study and laboratory tests, drafted the manuscript; NTN supervised laboratory tests, monitored laboratory quality and participated in the manuscript writing; SA, LK, EO, CA, MB performed patients enrollment, molecular analysis and participated in the manuscript writing. JPC has coordinated and helped to draft the manuscript. AM participated in the design of the study, coordination, and helped to draft the manuscript. All authors read and approved the final manuscript.

## References

[B1] Organisation mondiale de la Santé Surveillance de la résistance aux antipaludiques. Rapport d’une consultation de l’OMS, Genève, Suisse, 3–5 décembre 2001 2002 WHO/CDS/CSR/EPH/2002.17/WHO/CDS/RBM 39 http://whqlibdoc.who.int/hq/2002/who_cds_csr_eph_2002.17_fre.pdf

[B2] Aubouy A Fievet N Bertin G Sagbo JC Kossou H Kinde-Gazard D Kiniffo R Massougbodji A Deloron P Dramatically decreased therapeutic efficacy of chloroquine and sulfadoxine-pyrimethamine, but not mefloquine, in southern Benin Trop Med Int Health 2007 12 886 894 10.1111/j.1365-3156.2007.01859.x 17596256

[B3] Organisation mondiale de la Santé Evaluation et surveillance de l’efficacité des antipaludiques dans le traitement du paludisme à Plasmodium falciparum non compliqué 2003 OMS, Genève: Document WHO/RBM 50 67

[B4] Kyabayinze D Cattamanchi A Kamya MR Rosenthal PJ Dorsey G Validation of a simplified method for using molecular markers to predict sulfadoxine-pyrimethamine treatment failure in African children with falciparum malaria AmJTrop Med Hyg 2003 69 247 252 14628939

[B5] Kublin JG Dzinjalamala FK Kamwendo DD Malkin EM Cortese JF Martino LM Mukadam RA Rogerson SJ Lescano AG Molyneux ME Winstanley PA Chimpeni P Taylor TE Plowe CV Molecular markers for failure of sulfadoxine- pyrimethamine and chlorproguanil-dapsone treatment of *Plasmodium falciparum* malaria J Infect Dis 2002 185 380 388 10.1086/338566 11807721

[B6] Fidock DA Nomura T Talley AK Cooper RA Dzekunov SM Ferdig MT Ursos LM Sidhu AB Naudé B Deitsch KW Su XZ Wootton JC Roepe PD Wellems TE Mutations in the *P. falciparum* digestive vacuole transmembrane protein *Pf*CRT and evidence for their role in chloroquine resistance Mol Cell 2000 6 861 871 10.1016/S1097-2765(05)00077-8 11090624 PMC2944663

[B7] Sidhu AB Verdier-Pinard D Fidock DA Chloroquine resistance in *Plasmodium falciparum* malaria parasites conferred by *pfcrt* mutations Science 2002 298 210 213 10.1126/science.1074045 12364805 PMC2954758

[B8] Wellems TE Plowe CV Chloroquine-resistant malaria J Infect Dis 2001 184 770 776 10.1086/322858 11517439

[B9] Eberl KJ Jelinek T Aida AO Peyerl-Hoffmann G Heuschkel C El Valy AO Christophe EM Prevalence of polymorphisms in the dihydrofolate reductase and dihydropteroate synthetase genes of *Plasmodium falciparum* isolates from southern Mauritania Trop Med Int Health 2001 6 756 760 10.1046/j.1365-3156.2001.00791.x 11679122

[B10] Kublin JG Cortese JF Njunju EM Mukadam RA Wirima JJ Kazembe PN Djimdé AA Kouriba B Taylor TE Plowe CV Reemergence of chloroquine-sensitive *Plasmodium falciparum* malaria after cessation of chloroquine use in Malawi J Infect Dis 2003 187 1870 1875 10.1086/375419 12792863

[B11] Laufer MK Thesing PC Eddington ND Masonga R Dzinjalamala FK Takala SL Taylor TE Plowe CV Return of chloroquine antimalarialefficacy in Malawi N Engl J Med 2006 355 1959 1966 10.1056/NEJMoa062032 17093247

[B12] Nahum A Erhart A Ahounou D Bonou D Van Overmeir C Menten J Akogbeto M Coosemans M Massougbodji A D’Alessandro U Extended high efficacy of the combination sulphadoxine-pyrimethamine with artesunate in children with uncomplicated falciparum malaria on the Benin coast West Africa. Malar J 2009 8 37 10.1186/1475-2875-8-37 PMC2653068 19257898

[B13] Bertin G Briand V Bonaventure D Carrieu A Massougbodji A Cot M Deloron P Molecular markers of resistance to sulphadoxine pyrimethamine during intermittent preventive treatment of pregnant women in Benin Malar J 2011 10 196 10.1186/1475-2875-10-196 21767415 PMC3199903

[B14] Programme national de lutte contre le paludisme Plan stratégique de lutte contre le paludisme au Benin 2006–2010 http://www.rbm.who.int/countryaction/nsp/benin.pdf16614329

[B15] WHO/Communicable diseases cluster Severe falciparum malaria Trans R Soc Trop Med Hyg 2000 94 0S1 S90 11103309

[B16] Plowe CV Djimde A Bouare M Doumbo O Wellems TE Pyrimethamine and proguanil resistance-conferring mutations in *Plasmodium falciparum* dihydrofolate reductase: polymerase chain reaction methods for surveillance in Africa Am J TropMed Hyg 1995 52 565 568 10.4269/ajtmh.1995.52.565 7611566

[B17] Djimdé A Doumbo OK Cortese JF Kayentao K Doumbo S Diourté Y Coulibaly D Dicko A Su XZ Nomura T Fidock DA Wellems TE Plowe CV A molecular marker for chloroquine-resistant *falciparum* malaria N Engl J Med 2001 344 257 263 10.1056/NEJM200101253440403 11172152

[B18] Thomsen TT Ishengoma DS Mmbando BP Lusingu JP Vestergaard LS Theander TG Lemnge MM Bygbjerg IC Alifrangis M Prevalence of single nucleotide polymorphisms in the *Plasmodium falciparum* multidrug resistance gene (*Pfmdr-1*) in Korogwe District in Tanzania before and after introduction of artemisinin-based combination therapy Am J Trop Med Hyg 2011 85 979 983 10.4269/ajtmh.2011.11-0071 22144430 PMC3225174

[B19] Pearce R Drakeley C Chandramohan D Mosha F Roper C Molecular determination of point mutation haplotypes in the dihydrofolate reductase and dihydropteroate synthase of *Plasmodium falciparum* in three districts of Northern Tanzania Antimicrob Agents Chemother 2003 47 1347 1354 10.1128/AAC.47.4.1347-1354.2003 12654669 PMC152520

[B20] Akogbeto M Padonou GG Bankole HS Kinde Gazard D Gbedjissi GL Dramatic decrease in malaria transmission after large-scale indoor residual spraying with bendiocarb in Benin, an area of high resistance of *Anopheles gambiae* to pyrethroids Am J Trop Med Hyg 2011 85 586 593 10.4269/ajtmh.2011.10-0668 21976555 PMC3183760

[B21] Plowe CV Cortese JF Djimde A Nwanyanwu OC Watkins WM Winstanley PA Estrada-Franco JG Mollinedo RE Avila JC Cespedes JL Carterand D Doumbo OK Mutations in *Plasmodium falciparum* dihydrofolate reductase and dihydropteroate synthase and epidemiologic patterns of pyrimethamine-sulfadoxine use and resistance J Infect Dis 1997 176 1590 1596 10.1086/514159 9395372

[B22] Diourte Y Djimde A Doumbo OK Sagara I Coulibaly Y Dicko D Diallo M Diakite M Cortese JF Plowe CV Pyrimethamine-sulfadoxine efficacy and selection for mutations in *Plasmodium falciparum* dihydrofolate reductase and dihydropteroate synthase in Mali Am J Trop Med Hyg 1999 60 475 478 10466980 10.4269/ajtmh.1999.60.475

[B23] Doumbo OK Kayentao K Djimde A Cortese JF Diourte Y Konaré A Kublin JG Plowe CV Rapid selection of *Plasmodium falcipavum* dihydrofolate reductase mutants by pyrimethamine prophylaxis J Infect Dis 2000 182 993 996 10.1086/315787 10950805

[B24] Mula P Fernández-Martínez A de Lucio A Ramos JM Reyes F González V Benito A Berzosa P Detection of high levels of mutations involved in anti-malarial drug resistance in *Plasmodium falciparum* and *Plasmodium vivax* at a rural hospital in southern Ethiopia Malar J 2011 10 214 10.1186/1475-2875-10-214 21810256 PMC3161020

[B25] Mayengue PI Ndounga M Davy MM Tandou N Ntoumi F In vivo chloroquine resistance and prevalence of the pfcrt codon 76 mutation in *Plasmodium falciparum* isolates from the Republic of Congo Acta Trop 2005 95 219 225 10.1016/j.actatropica.2005.06.001 16002038

[B26] Bin Dajem SM Al-Farsi HM Al-Hashami ZS Al-Sheikh AA Al-Qahtani A Babiker HA Distribution of drug resistance genotypes in *Plasmodium falciparum* in an area of limited parasite diversity in Saudi Arabia AmJTrop Med Hyg 2012 86 782 788 10.4269/ajtmh.2012.11-0520 PMC3335680 22556074

[B27] Gesase S Gosling RD Hashim R Ord R Naidoo I Madebe R Mosha JF Joho A Mandia V Mrema H Mapunda E Savael Z Lemnge M Mosha FW Greenwood B Roper C Chandramohan D High Resistance of *Plasmodium falciparum* to Sulphadoxine/Pyrimethamine in Northern Tanzania and the emergence of *dhps* resistance mutation at codon 581 PLoS One 2009 4 e4569 10.1371/journal.pone.0004569 19238219 PMC2644264

[B28] Bouyou-Akotet MK Mawili-Mboumba DP Tchantchou TD Kombila M High prevalence of sulphadoxine/pyrimethamine-resistant alleles of *Plasmodium falciparum* isolates in pregnant women at the time of introduction of intermittent preventive treatment with sulphadoxine/pyrimethamine in Gabon J Antimicrob Chemother 2010 65 438 441 10.1093/jac/dkp467 20053688

[B29] Nzila A Mberu J Sulo H Dayo PA Winstanley CH Sibley W Watkins M Towards an understanding of the mechanism of pyrimethamine-sulfadoxine resistance in *Plasmodium falciparum:* genotyping of dihydrofolate reductase and dihydropteroate synthase of Kenyan parasites Antimicrob Agents Chemother 2000 44 991 996 10.1128/AAC.44.4.991-996.2000 10722502 PMC89803

[B30] Sibley CHJE Hyde PFG Plowe CV Kublin JG Mberu EK Cowman AF Winstanley PA Watkins WA Nzila AM Pyrimethamine-sulfadoxine resistance in *Plasmodium falciparum*: what next? Trends Parasitol 2001 17 582 588 10.1016/S1471-4922(01)02085-2 11756042

[B31] Tinto H Ouedraogo JB Zongo I Van Overmeir C Van Marck E Guiguemdé TR D’alessandro U Sulfadoxine-pyrimethamine efficacy and selection of *Plasmodium falciparum dhfr* mutations in Burkina Faso before its introduction as intermittent preventive treatment for pregnant women AmJTrop Med Hyg 2007 76 608 613 17426157

[B32] Mockenhaupt FP Teun BJ Eggelte TA Schreiber J Ehrhardt S Wassilew N Otchwemah RN Sauerwein RW Bienzle U *Plasmodium falciparum dhf*r but not *dhps* mutations associated with sulphadoxine-pyrimethamine treatment failure and gametocyte carriage in northern Ghana Trop Med Int Health 2005 10 901 908 10.1111/j.1365-3156.2005.01471.x 16135198

[B33] Frosch AEP Venkatesan M Laufer MK Patterns of chloroquine use and resistance in sub-Saharan Africa: a systematic review of household survey and molecular data Malar J 2011 10 116 10.1186/1475-2875-10-116 21554692 PMC3112453

[B34] Chippaux JP Massougbodji A Akogbeto M Josse R Zohoun T Sadeler BC Evolution de la chimiosensibilité de *Plasmodium falciparum* à la chloroquine et à la méfloquine au Benin entre 1980 et 1989 Bull Soc Path Exot 1990 83 320 329 2208463

[B35] Ginsburg H Famin O Zhang J Krugliak M Inhibition of glutathione-dependent degradation of heme by chloroquine and amodiaquine as a possible basis for their antimalarial mode of action Biochem Pharmacol 1998 56 1305 1313 10.1016/S0006-2952(98)00184-1 9825729

[B36] Holmgren G Gil JP Ferreira PM Veiga MI Obonyo CO Björkman A Amodiaquine resistant *Plasmodium falciparum* malaria *in vivo* is associated with selection of *pfcrt* 76T and *pfmdr1* 86Y Infect Gen Evol 2006 6 309 314 10.1016/j.meegid.2005.09.001 16271310

[B37] Zhang GQ Guan YY Zheng B Wu S Tang LH Molecular assessment of *Plasmodium falciparum* resistance to antimalarial drugs in China Trop Med Int Health 2009 14 1266 1271 10.1111/j.1365-3156.2009.02342.x 19772548

[B38] Kamugisha E Jing S Minde M Kataraihya J Kongola G Kironde F Swedberg G Efficacy of artemether-lumefantrine in treatment of malaria among under-fives and prevalence of drug resistance markers in Igombe-Mwanza, north-westernTanzania Malar J 2012 11 58 10.1186/1475-2875-11-58 22369089 PMC3305412

[B39] Raman J Mauff K Mulanga P Mussa A Maharaj R Barnes K Five years of antimalarial resistance marker surveillance in Gaza Province, Mozambique, following artemisinin-based combination therapy roll out PLoS One 2011 6 8 10.1371/journal.pone.0025992 PMC3195082 22022487

[B40] Pearce RJ Ord R Kaur H Lupala C Schellenberg J Shirima K Manzi F Alonso P Tanner M Mshinda H Roper C Schellenberg D A community-randomised evaluation of the effect of IPTi on anti-malarial drug resistance in southern Tanzania J Infect Dis 2013 207 848 859 10.1093/infdis/jis742 23225897

